# Food on the Move: The Impact of Implied Motion in Pictures on Food Perceptions through Anticipated Pleasure of Consumption

**DOI:** 10.3390/foods10092194

**Published:** 2021-09-16

**Authors:** Lana Mulier, Eva Meersseman, Iris Vermeir, Hendrik Slabbinck

**Affiliations:** Department of Marketing, Innovation and Organisation, Faculty of Economics and Business Administration, Ghent University, 9000 Ghent, Belgium; Eva.Meersseman@UGent.be (E.M.); Iris.Vermeir@UGent.be (I.V.); Hendrik.Slabbinck@UGent.be (H.S.)

**Keywords:** food pictures, motion perception, implied motion, food pleasure, visual advertising, replication study

## Abstract

To tackle obesity, upgrading the image of healthy food is increasingly relevant. Rather than focusing on long-term benefits, an effective way to promote healthy food consumption through visual advertising is to increase its pleasure perception. We investigate whether implied motion, a popular trend in food pictures, affects food perceptions through anticipated consumption pleasure. Prior research shows that motion affects food perceptions, but these studies focused on limited food categories, using experiments with a single food stimulus, and mainly showing unhealthy food effects. Therefore, we aim to (1) replicate prior findings on the effects of food in motion on appeal, tastiness, healthiness, and freshness perceptions; (2) examine whether these effects differ for healthy and unhealthy food; and (3) investigate whether anticipated pleasure of consumption drives the effects of implied motion on food perceptions. Three between-subjects experiments (*N* = 626) reveal no evidence for the effectiveness of motion (vs. no motion) across a large variety of food products. We further show no differential effects for healthy versus unhealthy foods. Moreover, implied motion does not increase appeal or taste perceptions through anticipated pleasure. Considering the current replication crisis, these findings provide more nuanced insights into the effectiveness of motion in visual food advertising.

## 1. Introduction

Visual food displays are omnipresent in our daily online and offline consumption environment. For instance, we encounter hamburgers on billboards while driving on the road, we scroll through pictures of salads and smoothies on Instagram, we see a pizza takeaway advertisement in a magazine or a food delivery application, we spot meal pictures on menus or the walls in a fast-food restaurant, and we select groceries through food photographs in an online grocery store. Because of this explosion of food-related visual content across traditional, digital, and social media, young generations and adults are continuously exposed to advertising containing food pictures [1,2,3].

Simultaneously, obesity rates are higher than ever. The World Health Organization estimated that a striking 39% of adults were overweight in 2016. Moreover, of these overweight people, 13% were obese. Increased consumption of food high on energy but low on nutrients is one of the reasons that lay at the basis of the obesity pandemic [4,5]. As such, obesity is preventable by decreasing unhealthy consumption [5]. Recent research shows that the perspective in which food is presented in pictures can help consumers in choosing less unhealthy food [6]. To diminish obesity rates, however, there is a need for not only lowering consumers’ intake of unhealthy food but, more importantly, also increasing their consumption of healthy food [7]. Unhealthy foods are tempting food products that do not imply long-term benefits on consumption, while healthy foods are not very tempting immediately but do have long-term benefits on body weight and health outcomes [8].

A contemporary and effective way to promote a healthy diet is to increase consumers’ pleasure perception of healthy food instead of solely focusing on its positive effects in the long run [9]. The latter has shown rather limited success [10,11]. Enhancing consumers’ healthy food pleasure perception means showing that consuming healthy food can be tasty, enjoyable, and exciting [12]. As such, a potentially efficient means to promote healthy food is using visual advertising techniques to increase pleasure perceptions.

Previous research has focused on the use of implied motion, a popular visual trend in food pictures, as an effective way to enhance food perceptions. Implied motion is the extraction of motion information from a static image [13], such as a picture of water being poured into a glass. According to Gvili et al. [14,15] and Gvili et al. [16], using implied motion to display food can increase evaluations of freshness, appeal, and projected taste of the food. These authors argue that food in motion (as opposed to food presented still) is evaluated more favorably, because of intuitive associations of movement with freshness. Moreover, Amar et al. [17] propose that food presented with implied motion can also increase the perceived healthiness of the food, through a similar enhanced perception of the freshness of the food.

However, these authors focused on limited food categories, using studies with a single food stimulus, and mainly showing effects for unhealthy foods. Today’s consumption environment, however, is packed with many different food displays fighting for consumers’ sparse attention. Furthermore, this previous research mainly examined the effects of motion for either unhealthy foods or healthy foods and did not consider potential differences in the effectiveness of motion between the two food categories.

Addressing these limitations, we investigate whether we can replicate the findings on the effectiveness of food in motion as shown by Amar et al. [17], Gvili et al. [14,15], and Gvili et al. [16]. Given the rising concerns over replicability in the social sciences [18], using an extensive and diverse set of food stimuli is imperative to test for the robustness and generalizability of the prior findings. Moreover, considering our inherent preference for unhealthy food over healthy food [19,20] and the growing obesity pandemic, a relevant objective is to examine whether implied motion in visual advertising works differently for healthy as opposed to unhealthy food promotion.

Furthermore, building on motion perception research, we propose that the use of implied motion in visual food presentation might be a way to increase the pleasure perception of food. Following the motion effect theory [21,22] and the vividness theory [23,24], motion in food pictures can evoke arousal and imagined consumption, which can increase consumers’ anticipated pleasure of consuming the food.

Therefore, the current research investigates the effectiveness of food in motion on food perceptions through an anticipated pleasure of consumption in three experimental studies. The aim of this research is threefold. First, we attempt to replicate the existing research on the effects of implied motion in food pictures on appeal and perceptions of tastiness, healthiness, and freshness. Secondly, we examine whether these effects differ for healthy and unhealthy food in motion. Thirdly, we aim to extend the findings on food in motion by investigating whether implied motion in pictures positively influences food perceptions through the anticipated pleasure of consuming the food. We test our objectives for a large variety of food stimuli and different samples and study contexts.

This research contributes to the existing literature in three ways. First, while prior research on food in motion shows that motion (versus no motion) can be an effective way to enhance the appeal, perceptions of taste, healthiness, and freshness, we do not find evidence for these effects across a large and varied set of food stimuli. Considering the current replication crisis, these findings provide more nuanced insights into how implied motion affects food perceptions. Secondly, we find no differences between healthy and unhealthy food in motion on food perceptions, although prior studies in food advertising suggest that visual cues can affect responses to healthy and unhealthy food differently. Thirdly, we investigate the relevant role of pleasure in healthy food promotion using implied motion. Our findings reveal that food in motion does not increase appeal or taste perceptions through an anticipated pleasure of consumption.

To the best of our knowledge, this study is the first extensive replication attempt of the effectiveness of food in motion. While many marketers often use implied motion in their visual advertising for food promotion, we show that the effectiveness of this practice is not straightforward. As such, we provide practical implications for food marketers, ad executives, and public policymakers.

### 1.1. The Strength of Visuals in Food Advertising

Consumers are constantly exposed to visual cues in today’s food environment, with its abundant availability of food at every part of the day [1,2,3]. Importantly, food selection and consumption are primarily guided by the visual system [25,26], as visual sensory information is salient, vivid, and readily available [27]. Previous research shows that the way food is visually presented can exert an impact on people’s subsequent food choices and their consumption behavior [25,28,29,30,31,32,33]. More specifically, visual elements can steer consumers’ behavior away from unhealthy foods or towards healthy foods. For example, when food pictures are shot from a top perspective (vs. a diner’s eye perspective), consumers choose less unhealthy food [6]. On the other hand, promoting healthy food in sensory advertising is found to be the most effective by visually presenting single-sense (vs. multiple-sense) elements [34].

Furthermore, visual aspects of a food product (e.g., packaging shape, picture of the food, logo, color of the food, etc.) guide consumers in forming expectations about the food’s quality, taste, healthiness, and other aspects [32]. The food quality properties that the majority of consumers value the most are freshness, taste, and healthiness [35,36,37]. Freshness can be defined as the closeness of the current state of the product to its original state in terms of sensory characteristics, distance, time, and processing [38]. Research shows that freshness is an important determinant for consumers’ food choices (e.g., for fruits and vegetables) [37]. Taste perception can be described as the sensations from taste buds with input from the other senses [39]. Research on taste perception suggests an automatic bottom-up process, meaning that information about the food is processed automatically and heuristically, driven by intrinsic or extrinsic aspects of the stimulus, such as the visual aspect [39].

Additionally, consumers are increasingly sensitive to the health qualities of the food they consume [40]. Perceived healthiness can be defined as a consumer’s expectation of a product’s influence on his or her state of health [41,42]. Health perceptions are also influenced by the visual aspect. For example, consumers perceive food products in a cold-colored (vs. warm-colored) packaging [43], and products in an angular (vs. rounded) packaging, as healthier [44]. Beliefs about the healthiness of food affect food consumption, as perceiving food as healthy increases intake of that food [45]. In today’s overload of food advertising, however, the perceived healthiness of products is not always easy to predict.

### 1.2. (Un)healthy Food Associations and the Concept of Food Pleasure

A common assumption in food research is that consumers want to make healthy food choices, but in reality, many consumers might be more worried about taste than the potential health benefits [46]. Research on the motivational processes underlying the conflict between healthiness and taste is sparse [42]. An important stream in food marketing research is the unhealthy = tasty intuition (UTI). This intuition suggests that food categorized as unhealthy results in higher taste perceptions than food categorized as healthy [39,47]. The support for this automatic, bottom-up process stems from the findings of an implicit association test (IAT), which shows that consumers are quicker to categorize unhealthy (vs. healthy) food as tasty [47]. The UTI thus is an explanation of the preference for highly caloric food, in the sense that consumers tend to overconsume food perceived as unhealthy because they spontaneously and automatically consider unhealthy food as tasting better than healthy food [48]. This research demonstrates that the use of extrinsic cues, in this case labeling a food item as either healthy or unhealthy, can have an automatic effect on taste perception [39]. This lay theory of the UTI can be explained by evolutionary research on human behavior. In the course of evolution, fatty and sugary foods played a vital role as sources of energy. People may have automatically perceived these food properties as tastier because they have proved effective in ensuring survival. People who considered these high-energy foods as tastier than low-energy foods had an evolutionary advantage [42,47,48].

However, the intuition that unhealthy food tastes better may vary cross-culturally. Specifically, because of strong intercultural differences in food perception, the UTI might only hold for US-American consumers, and not for French consumers [48]. In two studies, Werle et al. [48] show that the American unhealthy = tasty intuition does not exist in France, where an opposite healthy = tasty intuition prevails. This French intuition influences not only taste perceptions but also pleasure associated with food consumption and perceived product quality. Food pleasure orientation (FPO) is the general tendency of a person to associate eating with enjoyment and to generate pleasure from eating. Food is an important and positive part of consumers high in FPO, such as the French [49]. These people tend to focus more on the experience of eating and less on the health consequences of eating. Consequently, high FPO should increase anticipated pleasure of consuming a food product, which positively influences the perceived tastiness of the food and thus diminishes consumers’ healthy = less tasty intuition [50,51].

Moreover, advertisements can increase consumers’ anticipated food pleasure for a specific product. An advertisement can elicit positive food pleasure expectations by focusing on the taste and other pleasurable sensory aspects of a product [52]. This food pleasure approach is mostly used in advertisement efforts for unhealthy foods. However, for healthy food products, advertisements typically tend to focus on the healthy aspects of the product. Indeed, informing the consumers about the nutritional value and positive long-term health benefits plays a central role in these healthy food advertisements [12]. Unfortunately, this advertising angle has shown rather limited success in attracting consumers toward healthy food [10,11,53]. In addition to the healthy = not tasty intuition, healthy foods are often stigmatized as being boring, serious, and not exciting [54,55]. Therefore, to break these stigmas and attract more consumers towards healthy food options, advertisements promoting healthy foods could focus more on the tastiness and the pleasurable aspects of eating healthy [12]. Especially for consumers that eat rather unhealthy diets, these advertisements focusing on the pleasure of sharing, preparing, and eating healthy foods tend to be effective [9].

### 1.3. Motion Perception and Dynamic Imagery

We propose that the use of motion in visual presentations of food might be a way to increase the pleasure perception of healthy foods. Building on prior motion perception research, we can distinguish between real and implied motion [56,57]. Whereas real motion can be defined as the actual dynamic movement of objects or living organisms [56], implied motion is a snapshot of this dynamic movement. Following Kaiyun et al. [13], we define implied motion as the extraction of motion information from a stationary photo. These static images can freeze or capture the movement of a stimulus, which will result in dynamic motion imagery. In turn, viewers will form a mental representation of the action, which will ‘unfreeze’ the image again and complete the movement of the stimulus [58]. For example, when viewing a picture of coffee being poured into a mug, people can immediately detect and identify the movements of pouring liquid, even though they do not see the motion in real life.

Prior research has investigated dynamic motion imagery resulting from implied motion in the consumer behavior domain. For instance, Cian et al. [59] propose that static brand logos can evoke a perception of movement (i.e., dynamic imagery) and affect the level of consumer engagement, which then increases brand attitudes. This effect is moderated by the congruence between perceived movement and brand characteristics. As such, the authors argue that dynamic imagery is an important aspect of logo design, as it can enhance brand attitudes if used carefully.

Interestingly, neuroimaging studies have shown that exposure to implied motion activates similar regions of the brain as exposure to real motion [56,58,60,61,62]. Moreover, implied motion is predictive of fixation similarly to real motion [63]. In other words, people who view images with implied movement (e.g., pouring coffee), and those who watch short video fragments of the real movements should process the images in a similar way. As such, implied motion is commonly used in visual presentations of food.

### 1.4. The Use of Implied Motion in Food Pictures

When exploring the world’s largest online advertising archive [64], it is striking how many of the food advertisements contain implied motion. Some examples of popular print food advertisements of the United States are a bucket of steaming hash browns of Dunkin’ donuts, a dripping bottle of Vaughn’s BBQ sauce, and a breaking piece of Tillamook cheddar cheese. Furthermore, not only in print advertisements do we see this dynamic food picture trend but also on popular billboards. Think, for example, of billboards in the streets of the United Kingdom with chocolate icing running down a Mr. Kipling cake, or sauce dripping off of a taco with tofu of The Tofoo Co. Of course, this visual trend in food advertisements is commonly used in offline media as well as digital advertising. For instance, digital advertisements showed Narasu’s dripping and steaming coffee in India, and a bottle of chili Heinz ketchup in flames in Egypt. Next to commercial media, food pictures with implied motion are also very popular on social media. For instance, sprinkling sugar, pouring sauce, and oozing cheese are reoccurring themes in the Instagram pictures of the two most followed chefs, Jamie Oliver and Gordon Ramsay.

Prior research has investigated this use of implied motion in the food domain. A stream of studies shows that motion in food displays can increase food appeal [14,15,16,17,65]. A product that is shown moving is evaluated more favorably, due to associations of movement with freshness. Gvili et al. [15] and Gvili et al. [16] show that depictions of food with implied motion lead to enhanced evaluations of freshness, appeal, and projected taste of the food. This might be due to an overextension of a primitive link between motion and freshness, as the brain recognizes movement as an indicator of freshness and quality. By being sensitive to motion, people could judge the edibleness and freshness of food. For example, edible (vs. rotten) plants and fruits are hanging on living trees that will move in the wind, and moving (vs. non-moving) animals are more attractive and are judged to be healthier. Interestingly, even some inanimate foods are fresher when in motion [15], for example, running water is fresher than stagnant water because the latter enhances bacterial proliferation [66]. However, in our modern environment, this sensitivity to motion might extend to new settings where it is no longer relevant, thus creating an evolutionary trap [15,67,68]. For instance, whereas the natural link between motion and freshness was evident in the primitive environment, this link has lost its relevancy in today’s setting where food is instantly available [16]. As such, consumers intuitively associate food in motion with freshness—an association that has been reinforced by the common advertising practice of displaying mostly unhealthy food in motion.

Fortunately, implied motion could potentially help promote healthier food choices and consumption by increasing their appeal [15]. In two between-subjects experiments, Gvili and colleagues [15] exposed participants to a picture of a glass of orange juice that was either being poured (implied motion condition) or not (static condition). Participants’ appeal ratings of the juice were higher in the implied motion (vs. static) condition. The authors show that this effect is driven by an increased perception of freshness. In a follow-up study with four between-subjects experiments, Gvili and colleagues [16] further find that implied motion in food pictures of orange juice, pretzels, cereal, and yogurt also has a positive effect on taste expectations and that this is similarly mediated by perceived freshness. More recently, Amar, Gvili, and Tal [17] show that presenting food (yogurt, fish, and orange juice) with implied motion in advertisements can also improve the perceived healthiness of the food. Similarly, this effect is explained or mediated by the judgment of the freshness of the food.

However, the studies mentioned above involve several limitations. First, they include limited food categories and focus on a single food stimulus per experiment. Would these findings still hold when viewing multiple food stimuli, as is the case in our daily consumption environment? Our visual system is overloaded with food displays fighting for our sparse attention. In order to exclude the influence of idiosyncratic effects, testing many randomized food stimuli (i.e., both solid and liquid foods, pouring and dropping motions, plain and more detailed food presentations, etc.) across experiments would increase the robustness and generalizability of the findings. Secondly, the existing studies mainly focused on unhealthy food products (e.g., pretzels and cereal) and did not differentiate the findings per food category. However, considering the inherent human preference for unhealthy food over healthy food [19,20], it might be possible that implied motion in food pictures works differently for healthy as opposed to unhealthy food presentation. Furthermore, one of the limited healthy stimuli used in the experiments is a living and swimming fish [17], rather than a fish ready to be eaten on a plate. One could argue that this is not an optimal choice to depict implied motion versus no motion as even the fish that is not jumping out of the water still is a living creature in motion. Addressing these limitations, we investigate whether we can replicate and extend the findings on the effectiveness of food in motion as shown by Amar et al. [17], Gvili et al. [15], and Gvili et al. [16].

### 1.5. Research Aims and Hypotheses

The ability to replicate previous research is essential for assessing the credibility of scientific findings [18]. Throughout the last decade, the so-called replication crisis in the social sciences has stimulated many researchers to conduct large-scale replication projects (e.g., [18,69,70,71]). The low rate of replication success in some of these projects has led to an increased awareness of the methodological crisis and an increased interest in research on the scientific process of replication itself (e.g., [18,72]). Considering this replication crisis, it is imperative to test the effects of implied motion on food perceptions across multiple studies and food products. As we use different methods, stimuli, and measures compared to the prior studies, we consider our studies conceptual replications, extensions, or generalizability studies, rather than exact replications [73].

We first propose five main effects hypotheses for the effects of food in motion on food perceptions (H1–H5). Following the findings of Gvili et al. [15], Gvili et al. [16], and Amar et al. [18], we state four replication hypotheses for the effect of food in motion (vs. static food) on appeal and perceptions of taste, healthiness, and freshness, respectively:

**Hypothesis** **1** **(H1).** *Food in motion will be rated as more appealing than static food.*

**Hypothesis** **2** **(H2).** *Food in motion will be rated as tastier than static food*.

**Hypothesis** **3** **(H3).** *Food in motion will be rated as healthier than static food*.

**Hypothesis** **4** **(H4).** *Food in motion will be rated as fresher than static food*.

Further, we argue that implied motion in food pictures can increase the perception of pleasure of eating the food. The motion effect theory shows that motion in pictures increases arousal, as evidenced by an increase in skin conductance and a change in heart rate [21,22]. Moreover, the vividness effect theory suggests that motion is a vivid cue, which is emotionally interesting, concrete, imagery provoking, and proximate in a sensory, temporal, or spatial way [23,24]. As such, because of the arousal and potentially imagined consumption resulting from implied motion in food pictures, we reason that food in motion will increase consumers’ anticipated pleasure of consuming the food. As a fifth main effect hypothesis, we propose that:

**Hypothesis** **5** **(H5).** *Food in motion will evoke a higher anticipated pleasure of consumption compared to static food*.

These five main effects hypotheses will further be tested for both healthy and unhealthy food in motion. Based on the prior findings on food in motion, we do not expect particular differences in the effectiveness of healthy versus unhealthy food in motion, and as such we do not formulate specific hypotheses.

Next, we formulate two mediation effects hypotheses. Following the previous findings on the underlying process for the effects of motion on food perceptions, we propose a replication hypothesis for the mediating effect of freshness perception on appeal [15], perceived taste [16], and perceived healthiness [18], respectively. More formally:

**Hypothesis** **6** **(H6).** *Perceived freshness of the food will mediate the positive effect of motion (vs. no motion) on (a) appeal, (b) perceived taste, and (c) perceived healthiness of the food*.

We further examine the role of food pleasure as an alternative underlying process for the effects of motion on food perceptions. We investigate pleasure anticipation of consuming food as a state variable evoked by the depiction of food with implied motion. Following the vividness effect theory, motion is a vivid cue that is more appealing than no motion [23]. As pleasure perceptions of food can increase the appeal and projected taste of the food [12,50,51], we propose that:

**Hypothesis** **7** **(H7).** *Anticipated pleasure of consumption will mediate the positive effect of motion on (a) appeal, and (b) perceived taste of the food*.

Finally, we also examine the role of food pleasure as a trait variable, in terms of a person’s food pleasure orientation (FPO). Building on prior research on the FPO [50,51], we argue that consumers high in FPO will express a higher anticipated pleasure of consuming food presented in motion (vs. no motion) compared to consumers low in FPO. This perception of pleasure will then increase the appeal and perceived taste of the food in motion. In sum, we propose that:

**Hypothesis** **8** **(H8).** *The indirect effect of food in motion (vs. static food) on (a) appeal, and (b) perceived taste, through the anticipated pleasure of consumption, will be stronger for people with a high (vs. low) food pleasure orientation*.

**Figure 1:**
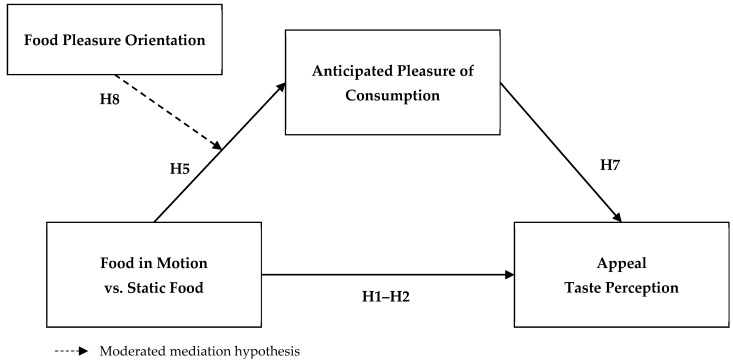
Conceptual model of the hypotheses (excluding some of the replication hypotheses).

Our conceptual model of the hypotheses is displayed in Figure 1.

## 2. Study Overview

In three experimental studies (total *N* = 626), we investigate the effects of implied motion in food pictures on appeal, perceptions of tastiness, healthiness, freshness, and anticipated pleasure of consumption. We use an extensive and diverse set of experimental stimuli (a total of 56 pretested food pictures) and different samples (undergraduates and older participants) and study contexts (studies conducted in the lab and online) to test for the robustness and generalizability of the findings. An overview of the studies can be found in Table 1.

To ensure an adequate sample size for each study a priori, we first performed a power analysis using G*Power [74]. Cohen’s *d* effect sizes from the 10 experimental studies on the effects of food in motion as reported in [15,16,17] varied between 0.396 and 1.771, resulting in an average Cohen’s *d* of 0.763, suggesting a medium to large effect size. The average total sample size in these studies was 70 participants. For our studies, considering an ANCOVA analysis with an alpha of 0.05, 3 covariates, and an expected effect size Cohen’s *f* of 0.382 (medium to large effect size, based on the Cohen’s *d* of 0.763), a minimum of 92 participants was required for a power of 0.95. However, the original studies may be underpowered given the low average sample size. To safeguard against a potential overestimation of the effects in their analyses, we implemented the safeguard power analysis method by [75]. This method is based on the lower 60% confidence interval of the target effect size, to protect against smaller true underlying effects. The minimum required sample size corresponding with an original effect size of *d* = 0.8 for a power of 0.95 and an alpha of 0.05 was 146 participants [75]. All three studies were conducted in accordance with the Declaration of Helsinki for Research involving Human Subjects and received approval from the Ethics Committee of the Faculty of Economics and Business Administration, Ghent University. All participants gave their informed consent for inclusion before participating in a study or pretest. All data for each study were analyzed using IBM SPSS Statistics 25 (IBM Corp., Armonk, NY, USA).

## 3. Study 1

In the first study, we tested the hypotheses regarding the main effects of food in motion on appeal, perceptions of taste, healthiness, freshness, and anticipated pleasure (H1–H5). Furthermore, we tested the hypotheses about the mediation effects of both freshness (H6) and anticipated pleasure (H7). Finally, we tested the moderated mediation hypothesis by looking at the interaction between food in motion and FPO (H8).

### 3.1. Stimuli and Pretest

The experimental stimuli for Study 1 and Study 2a were created by selecting a range of 36 relevant food pictures displaying implied motion in online, publicly available photo databases (i.e., a variety of liquid and solid foods as well as healthy and unhealthy foods). To create the static food condition, we manipulated each motion picture by erasing the implied movements from the picture (e.g., pouring or squeezing liquid and sauce, dropping or sprinkling food particles, etc.) with professional photo editing software.

We conducted a pretest to find which of the foods in the pictures were perceived as either healthy or unhealthy (relevant for Study 2a), but at the same time to verify whether the manipulated pictures were sufficiently appealing and realistic. A total of 122 participants (48% men, *M*_age_ = 25 years; *SD* = 6.36) were recruited for this pretest, which was included in a 50-min lab session of multiple unrelated studies. Participants were undergraduates, who took part in the lab session in return for a course credit, and paid participants, who received eight euros for completion of the session. Following a short introduction, pretest participants were presented with 36 randomized food pictures in the static condition. After exposure to each food picture, participants were asked to indicate how healthy they perceived the food to be, and how appealing and realistic they perceived the picture to be on 9-point Likert scales (1 = Not at all, 9 = Very much). Afterward, participants filled out their gender and birth year.

For each food picture, we ran a series of three One-Sample T-Tests (one per measure), using the average value of the scale (i.e., 5) as the test value. For this study, 26 food pictures (Table A1) that were rated as sufficiently appealing (*M* = 5.92, *SD* = 2.16) and realistic (*M* = 6.37, *SD* = 2.00), served as our experimental stimuli. These pictures consisted of 14 foods that were perceived as healthy (*M* = 7.64, *SD* = 1.68; i.e., salad, Brussels sprouts, limes, lemons, olive oil, honey, water, milk, tea, coffee beans, orange juice, and tomato juice) and 12 foods that were perceived as unhealthy (*M* = 3.43, *SD* = 1.61; i.e., cake, pancakes, muffin, coffee, lemonade, grenadine, red wine, and beer; Table 2).

### 3.2. Participants

A total of 159 participants (57% men, *M*_age_ = 25 years; *SD* = 4.72) took part in this laboratory study, which was included in a 50-min lab session of multiple unrelated studies. Participants were mostly undergraduates who participated in return for a course credit, and paid participants who received eight euros for completion of the session. We used two attention checks in the experiment to screen the data after data collection by including an item in the questionnaires for the Food Pleasure Orientation Scale and the Health Consciousness Scale (HCS), asking “*Please indicate ‘rather disagree’ for this statement*”. All participants answered these statements correctly and thus there were no exclusions from the dataset. They were randomly assigned to one of two between-subjects conditions: food in motion (*n* = 79) or static food (*n* = 80).

### 3.3. Procedure, Measurements, and Reliability

Participants were first informed that this study was about viewing and evaluating food pictures and were then exposed to 26 randomized food stimuli, presented either in motion or static, depending on the condition they were assigned to. After exposure to each picture, participants were first asked to rate the food presented in the picture (e.g., cake) on a series of characteristics on 9-point Likert scales (1 = Not at all, 9 = Very much), being the appeal, taste perception, healthiness perception, and freshness perception. Secondly, we measured anticipated pleasure of consumption with a one-item 9-point Likert scale, asking: “*To what extent do you anticipate pleasure in consuming this food (e.g., cake)?*” (1 = Not at all, 9 = Very much).

After stimulus presentation, we assessed participants’ food pleasure orientation as a trait variable, using the FPO Scale [50,76]. Participants reported their agreement with 6 items on a 7-point Likert scale (1 = Strongly disagree, 7 = Strongly agree), being (1) “*Enjoying food is one of the most important pleasures in my life*”; (2) “*I would rather eat my favorite meal than watch my favorite television show*”; (3), “*I think about food in a positive anticipatory way”*; (4) “*Money spent on food is money well spent*”; (5) “*I have fond memories of family food occasions*”; and (6) *‘‘If I could satisfy my nutritional needs safely, cheaply and without hunger by taking a daily pill, I would do this*”. The final item was reverse coded and the item scores were averaged so that a higher score indicated higher food pleasure orientation (*M* = 5.25; α = 0.54, suggesting low internal consistency reliability of this scale, but no items could be removed to improve Cronbach’s Alpha).

Further, participants’ evaluations of healthy and unhealthy food pictures could be affected by their health consciousness (HC), which is the extent to which an individual tends to undertake health actions [77]. These actions include greater concerns to health, caring about health, engaging in searching for health information, and valuing healthy conditions [78,79]. Using the HCS [80], participants reported their agreement with nine items on a 7-point Likert scale (1 = Strongly disagree, 7 = Strongly agree; e.g., “*I reflect about my health a lot*”). The item scores were averaged so that a higher score indicated higher health consciousness (*M* = 4.84; α = 0.93). We included this trait variable as a potential covariate in our analyses. Next, we asked participants to indicate how hungry and thirsty they felt during the study, on 7-point Likert scales (1 = Not hungry/thirsty at all, 7 = Very hungry/thirsty). As the experimental pictures involve a variety of foods and drinks, we included participants’ hunger and thirst levels as potential covariates in our analyses. Finally, participants filled out their gender and birth year.

### 3.4. Data Analysis

Because the dataset comprised results on multiple stimuli viewed by each participant, we ran multilevel analyses taking the hierarchical data structure into account, using the restricted maximum likelihood (REML) estimation technique and variance components (VC) as the default covariance structure. We estimated the intercept and slope randomly in each analysis to account for participant-level and stimulus-level effects. Starting from fixed-effects models, model fit improved when allowing for a random intercept, but it did not improve further when we allowed for a random slope estimation.

To compare the appeal, perceptions of taste, healthiness, freshness, and anticipated pleasure between food in motion and static food (H1–H5), we conducted five multilevel analyses with these five food perceptions as dependent measures respectively, motion (0 = Static food, 1 = Food in motion) as the independent variable, the 26 food stimuli as the within-subjects variable, and participants’ HC and hunger and thirst levels as covariates.

Further, we ran three multilevel mediation analyses via MLmed [81] to test whether perceived freshness mediates the effect of food in motion (vs. static food) on appeal, taste perception, and healthiness perception (H6). In these analyses, motion served as the independent variable, freshness perception as the mediator, appeal, taste perception, and healthiness perception as dependent measures, respectively, and participants’ HC, hunger, and thirst levels as covariates. Additionally, we ran two multilevel mediation analyses via MLmed to test whether anticipated pleasure mediates the effect of food in motion (vs. static food) on appeal and taste perception (H7). Similarly, motion served as the independent variable, anticipated pleasure as the mediator, appeal and taste perception as dependent measures, respectively, and participants’ HC, hunger and thirst levels as covariates.

Finally, we ran two multilevel moderated mediation analyses via MLmed [82,83], to test whether the indirect effect of food in motion (vs. static food) on appeal and perceived taste, respectively, through the anticipated pleasure of consumption will be stronger for people with a high (vs. low) FPO (H8). Motion served as the independent variable, FPO as the moderator, anticipated pleasure as the mediator, appeal and taste perception as dependent measures, respectively, and participants’ HC, hunger and thirst levels as covariates.

### 3.5. Results and Discussion

#### 3.5.1. Main Effects Analyses

**Appeal (H1).** The effect of motion on appeal was not significant (*F*(1, 154) = 1.48, *p* = 0.226, *d* = 0.194). Participants did not indicate a higher appeal of food in motion (*M* = 6.16, *SD* = 0.65) compared to static food (*M* = 6.00, *SD* = 0.84).

**Taste perception (H2).** The effect of motion on taste perception was not significant (*F*(1, 154) = 0.29, *p* = 0.594, *d* = 0.086). Participants did not perceive food in motion as tastier (*M* = 6.07, *SD* = 0.68) than static food (*M* = 5.99, *SD* = 0.87).

**Healthiness perception (H3).** The effect of motion on healthiness perception was not significant (*F*(1, 154) = 0.74, *p* = 0.390, *d* = 0.137). Participants did not perceive food in motion as healthier (*M* = 5.47, *SD* = 0.55) than static food (*M* = 5.54, *SD* = 0.66).

**Freshness perception (H4).** The effect of motion on freshness perception was not significant (*F*(1, 154) = 0.45, *p* = 0.506, *d* = 0.107). Participants did not perceive food in motion as more fresh (*M* = 6.15, *SD* = 0.71) than static food (*M* = 6.05, *SD* = 0.76).

**Anticipated pleasure of consumption (H5).** The effect of motion on anticipated pleasure was not significant (*F*(1, 154) = 0.23, *p* = 0.630, *d* = 0.077). Participants did not indicate a higher anticipated pleasure of consuming food in motion (*M* = 5.90, *SD* = 0.71) compared to static food (*M* = 5.81, *SD* = 0.89).

**Effects of the covariates.** Participants’ HC had a significant effect on appeal (*F*(1, 154) = 5.12, *p* = 0.025, *d* = 0.361). The higher participants’ HC, the lower they rated the appeal of the food (*r* = −0.16, *p* = 0.050). Participants’ hunger level significantly influenced anticipated pleasure (*F*(1, 154) = 3.33, *p* = 0.070, *d* = 0.291), meaning that the hungrier participants felt during the study, the higher their anticipated pleasure of consumption (*r* = 0.19, *p* = 0.019). Participants’ thirst level significantly influenced appeal (*F*(1, 154) = 10.35, *p* = 0.002, *d* = 0.514), taste perception (*F*(1, 154) = 3.94, *p* = 0.049, *d* = 0.317), and anticipated pleasure (*F*(1, 154) = 5.89, *p* = 0.016, *d* = 0.387). That is, the thirstier participants felt during the study, the higher they rated the appeal (*r* = 0.24, *p* = 0.003), tastiness (*r* = 0.17, *p* = 0.033), and anticipated pleasure of consuming the food (*r* = 0.22, *p* = 0.006).

This study reveals non-significant findings for all five main effects hypotheses (H1–H5; Table 3). Contrary to earlier findings [14,15,16,17], we find that food in motion does not evoke a higher appeal (H1), taste perception (H2), healthiness perception (H3), or freshness perception (H4) compared to static food. As such, Study 1 could not replicate the results from prior studies on the effectiveness of implied motion in pictures on food perceptions. Moreover, in contrast to what we expected in H5, food in motion (vs. static food) does not increase anticipated pleasure of consuming the food.

Additionally, separate analyses for foods and drinks showed no significant effects of motion (vs. no motion) on any of the food perception variables in line with the full analyses outlined here, which is why we do not report these further.

#### 3.5.2. Mediation and Moderated Mediation Analyses

**Mediation of freshness perception (H6).** According to the multilevel mediation analyses (model 4; [83]) with 10,000 Monte Carlo samples and 95% bias-corrected intervals (CIs), the effect of motion on freshness perception was not significant (*B* = 0.08, *SE* = 0.12, *t*(154) = 0.67, *p* = 0.506, *d* = 0.106). We found significant effects of freshness perception on appeal (*B* = 0.65, *SE* = 0.06, *t*(153) = 10.65, *p* < 0.001, *d* = 1.689), taste perception (*B* = 0.49, *SE* = 0.08, *t*(153) = 6.55, *p* < 0.001, *d* = 1.039), and healthiness perception (*B* = 0.54, *SE* = 0.05, *t*(153) = 10.36, *p* < 0.001, *d* = 1.634). The tests of mediation revealed no significant indirect effect of motion through freshness perception on appeal (ab = 0.05, *SE* = 0.08, 95% CI = (−0.1008; 0.2039)), taste perception (ab = 0.04, *SE* = 0.06, 95% CI = (−0.0736; 0.1556)), and healthiness perception (ab = 0.04, *SE* = 0.06, 95% CI = (−0.0824; 0.1715). The remaining direct effects of motion on appeal (*B* = 0.09, *SE* = 0.09, *t*(153) = 1.03, *p* = 0.306, *d* = 0.163) and taste perception (*B* = 0.03, *SE* = 0.11, *t*(153) = 0.25, *p* = 0.802, *d* = 0.04) were not significant, but there was a marginally significant remaining direct effect of motion on healthiness perception (*B* = −0.13, *SE* = 0.08, *t*(153) = −1.68, *p* = 0.096, *d* = −0.266). Among the covariates, HC (*B* = −0.09, *SE* = 0.04, *t*(153) = −1.99, *p* = 0.049, *d* = −0.316) and thirst level (*B* = 0.10, *SE* = 0.03, *t*(153) = 2.91, *p* = 0.004, *d* = 0.462) were significantly related to appeal, but hunger level was not significantly related to any dependent measure (*p* > 0.05).

These results show that a higher perceived freshness of food affects the (a) appeal, (b) perceived taste, and (c) healthiness of the food, but it does not mediate the effects of motion (vs. no motion) on these food perceptions. As such, these findings do not provide support for H6 nor replicate the results from prior studies on the mediating effects of freshness perception for the effect of implied motion on food perceptions [14,15,16,17].

**Mediation of anticipated pleasure of consumption (H7).** According to the multilevel mediation analyses (model 4; [83]) with 10,000 Monte Carlo samples and 95% CIs, the effect of motion on anticipated pleasure was not significant (*B* = 0.06, *SE* = 0.13, *t*(154) = 0.48, *p* = 0.630, *d* = 0.076). We found significant effects of anticipated pleasure on appeal (*B* = 0.70, *SE* = 0.05, *t*(153) = 14.25, *p* < 0.001, *d* = 2.25), and taste perception (*B* = 0.89, *SE* = 0.03, *t*(153) = 26.28, *p* < 0.001, *d* = 4.168). The tests of mediation revealed no significant indirect effect of motion through anticipated pleasure on appeal (ab = 0.04, *SE* = 0.09, 95% CI = (−0.1303; 0.2141)), and taste perception (ab = 0.05, *SE* = 0.11, 95% CI = (−0.1661; 0.2690)). The remaining direct effects of motion on appeal (*B* = 0.10, *SE* = 0.08, *t*(153) = 1.30, *p* = 0.197, *d* = 0.206) and taste perception (*B* = 0.01, *SE* = 0.05, *t*(153) = 0.23, *p* = 0.819, *d* = 0.036) were not significant. Among the covariates, HC (*B* = −0.08, *SE* = 0.04, *t*(153) = −2.03, *p* = 0.045, *d* = −0.322) and thirst level (*B* = 0.06, *SE* = 0.03, *t*(153) = 2.07, *p* = 0.041, *d* = 0.328) were significantly related to appeal, and hunger level was marginally significantly related to appeal (*B* = −0.04, *SE* = 0.02, *t*(153) = −1.92, *p* = 0.056, *d* = −0.305).

While we find that anticipated pleasure of consuming food increases the (a) appeal and (b) taste perception of the food, it does not mediate the effect of motion (vs. no motion) on these food perceptions. As such, we do not find evidence in support of H7.

**Moderated mediation of FPO (H8).** We ran two multilevel moderated mediation analyses (model 7; [82]), with 10,000 Monte Carlo samples, 95% CIs, and moderator FPO mean-centered at 5.25. The effect of FPO on anticipated pleasure was significant (*B* = 0.31, *SE* = 0.11, *t*(152) = 2.73, *p* = 0.007, *d* = 0.433), but the interaction effect of motion and FPO on anticipated pleasure was not (*B* = −0.20, *SE* = 0.16, *t*(152) = −1.26, *p* = 0.209, *d* = −0.2). There was no significant index of moderated mediation through anticipated pleasure on appeal (ab = −0.14, 95% CI = (−0.3727; 0.0793)) nor on taste perception (ab = −0.18, 95% CI = (−0.4705; 0.1065)).

As such, although we find that people with a higher (vs. lower) FPO indicate a higher anticipated pleasure of food consumption, the interaction effects of motion and FPO on (a) appeal, and (b) taste perception are not mediated by anticipated pleasure of consumption. These results do not provide support for H8. The indirect effect of food in motion (vs. static food) on appeal and perceived taste, through the anticipated pleasure of consumption, is not moderated by people’s food pleasure orientation.

## 4. Study 2a

In Study 2a–b, we again tested the main effects hypotheses of food in motion on food perceptions (H1–H5). However, different from Study 1, to disentangle potential differences between healthy and unhealthy food in motion, we created an additional between-subjects condition of food category (healthy vs. unhealthy foods) in the experimental design of these two studies. Hence, in contrast to Study 1, to make an explicit difference in the food category, we used very clear and unambiguous examples of healthy and unhealthy food (as indicated by the pretest, see Table A1). We further tested our mediation effects hypotheses of freshness (H6) and anticipated pleasure (H7), as well as the moderated mediation hypothesis of FPO (H8).

### 4.1. Stimuli and Pretest

The experimental stimuli for Study 2a were taken from the set of food pictures pretested in Study 1 (Table A1; nine stimuli were the same as in Study 1). Based on the results from the One-Sample T-Tests, we selected a total of 18 food pictures, consisting of 9 foods that were perceived as healthy (*M* = 7.82, *SD* = 1.26; i.e., salad, Brussels sprouts, water, tea, tomato juice, granola, muesli, smoothie, and fruit salad) and 9 foods that were perceived as unhealthy (*M* = 2.50, *SD* = 1.39; i.e., cake, pancakes, donuts, muffin, ice cream, lemonade, and beer; Table 4). Similar to Study 1, the food pictures were rated as sufficiently appealing (*M* = 6.53, *SD* = 1.89) and realistic (*M* = 6.18, *SD* = 1.81).

### 4.2. Participants

A total of 261 participants (44% men, *M*_age_ = 33 years; *SD* = 13.76) completed this study and were recruited online via our consumer panel. Analogous to Study 1, we used two attention checks in the experiment to screen the data after data collection. Specifically, we included an item in the FPO Scalethat asked participants to “*Please indicate ‘rather disagree’ for this statement*”, and another item in the HCS, asking them to “*Please indicate ‘neutral’ for this statement*”. Ten participants failed to answer these statements correctly and were excluded from the dataset. Our final sample included 251 participants who were randomly assigned to one of four between-subjects conditions: healthy food in motion (*n* = 63), unhealthy food in motion (*n* = 65), healthy static food (*n* = 56), or unhealthy static food (*n* = 67).

### 4.3. Procedure, Measurements, and Reliability

Participants received a similar introduction as in Study 1, and were asked to indicate how hungry and thirsty they currently felt on 7-point Likert scales (1 = Not hungry/thirsty at all, 7 = Very hungry/thirsty). We again included participants’ hunger and thirst levels as potential covariates in our analyses, but we now measured these control variables before stimulus presentation. Participants were then exposed to nine randomized healthy or unhealthy food stimuli, presented either in motion or static, depending on the condition they were assigned to. After exposure to each picture, participants answered the same set of questions. First, and similar to Study 1, participants rated the appeal, taste perception, healthiness perception, and freshness perception of the food presented in the picture (e.g., salad) on 9-point Likert scales (1 = Not at all, 9 = Very much). Secondly, we measured anticipated pleasure of consumption with a one-item 9-point Likert scale, using slightly different wording than in Study 1. Participants were asked: “*How much pleasure do you anticipate in consuming this food [e.g., salad]?*” (1 = No pleasure at all, 9 = A lot of pleasure).

After stimulus presentation, we assessed participants’ FPO in the same way as in Study 1, using the FPO Scale [50,76] on a 7-point Likert scale (1 = Strongly disagree, 7 = Strongly agree; *M* = 5.29; α = 0.70). Similarly, we assessed participants’ HC using the HCS [80] on a 7-point Likert scale (1 = Strongly disagree, 7 = Strongly agree; *M* = 4.98; α = 0.92). Again, we included HC as a potential covariate in our analyses. Finally, participants filled out their gender and birth year.

### 4.4. Data Analysis

We ran multilevel analyses taking the hierarchical data structure into account, using the REML estimation technique, VC as the default covariance structure, and estimating the intercept and slope randomly to account for participant-level and stimulus-level effects. Starting from fixed-effects models, model fit improved when we allowed for a random intercept, but it did not improve further when allowing for a random slope estimation.

The hypothesis testing for Study 2a is similar to Study 1, except for the main effects testing of food in motion (H1–H5). Specifically, we added the effect of healthy versus unhealthy food as a second main effect, as well as an interaction effect between motion (food in motion vs. static food) and food category (healthy vs. unhealthy food). These tests aim to assess potential differences in the effects of implied motion on perceptions of healthy versus unhealthy foods. To test the main effects as proposed in H1–H5, we conducted five multilevel analyses with appeal, taste perception, healthiness perception, freshness perception, and anticipated pleasure as dependent measures, respectively, motion (0 = Static food, 1 = Food in motion), food category (0 = Unhealthy food, 1 = Healthy food), and their interaction as independent variables, the nine food stimuli as the within-subjects variable, and participants’ HC, hunger, and thirst levels as covariates.

Further, we ran three multilevel mediation analyses via MLmed [81] to test H6. Motion served as the independent variable, freshness perception as the mediator, appeal, taste perception, and healthiness perception as dependent measures, respectively, and participants’ HC, hunger, and thirst levels as covariates. Additionally, we ran two multilevel mediation analyses via MLmed (testing H7), with motion as the independent variable, anticipated pleasure as the mediator, appeal and taste perception as dependent measures, respectively, and participants’ HC and hunger and thirst levels as covariates.

Finally, we ran two multilevel moderated mediation analyses via MLmed [82,83], to test H8. Motion served as the independent variable, FPO as the moderator, anticipated pleasure as the mediator, appeal and taste perception as dependent measures, respectively, and participants’ HC, hunger, and thirst levels as covariates.

### 4.5. Results and Discussion

#### 4.5.1. Main Effects and Interaction Effects Analyses

**Appeal (H1).** The effect of motion on appeal was not significant (*F*(1, 244) = 0.70, *p* = 0.403, *d* = 0.106). Participants did not indicate a higher appeal of food in motion (*M* = 6.13, *SD* = 1.47) compared to static food (*M* = 5.95, *SD* = 1.43). The effect of food category on appeal was significant (*F*(1, 244) = 54.64, *p* < 0.001, *d* = 0.938). Participants indicated a higher appeal of healthy food (*M* = 6.67, *SD* = 1.15) compared to unhealthy food (*M* = 5.47, *SD* = 1.45). There was no significant interaction effect between motion and food category (*F*(1, 244) = 0.00, *p* = 0.982, *d* = 0). There was no difference in appeal between healthy food in motion (*M* = 6.71, *SD* = 1.27) and healthy static food (*M* = 6.63, *SD* = 1.01; *t*(244) = 0.56, *p* = 0.577, *d* = 0.103), or between unhealthy food in motion (*M* = 5.56, *SD* = 1.43) and unhealthy static food (*M* = 5.38, *SD* = 1.48; *t*(244) = 0.62, *p* = 0.533, *d* = 0.108).

**Taste perception (H2).** The effect of motion on taste perception was not significant (*F*(1, 244) = 0.13, *p* = 0.717, *d* = 0.046). Participants did not perceive food in motion as tastier (*M* = 5.78, *SD* = 1.35) than static food (*M* = 5.71, *SD* = 1.34). The effect of food category on taste perception was significant (*F*(1, 244) = 31.30, *p* < 0.001, *d* = 0.71). Healthy food was perceived as tastier (*M* = 6.21, *SD* = 1.11) than unhealthy food (*M* = 5.33, *SD* = 1.40). There was no significant interaction effect between motion and food category (*F*(1, 244) = 0.04, *p* = 0.837, *d* = 0.004). Perceived taste did not differ between healthy food in motion (*M* = 6.19, *SD* = 1.20) and healthy static food (*M* = 6.23, *SD* = 1.01; *t*(244) = 0.11, *p* = 0.915, *d* = 0.02), or between unhealthy food in motion (*M* = 5.40, *SD* = 1.37) and unhealthy static food (*M* = 5.27, *SD* = 1.43; *t*(244) = 0.41, *p* = 0.679, *d* = 0.071).

**Healthiness perception (H3).** The effect of motion on healthiness perception was not significant (*F*(1, 244) = 0.92, *p* = 0.339, *d* = 0.122). Participants did not perceive food in motion as healthier (*M* = 5.12, *SD* = 2.63) than static food (*M* = 4.81, *SD* = 2.69). The effect of food category on healthiness perception was significant (*F*(1, 244) = 1720.31, *p* < 0.001, *d* = 5.264). Not surprisingly, healthy food was perceived as healthier (*M* = 7.58, *SD* = 0.72) than unhealthy food (*M* = 2.61, *SD* = 1.11). There was no significant interaction effect between motion and food category (*F*(1, 244) = 0.39, *p* = 0.531, *d* = 0.008). Perceived healthiness did not differ between healthy food in motion (*M* = 7.60, *SD* = 0.76) and healthy static food (*M* = 7.55, *SD* = 0.66; *t*(244) = 0.22, *p* = 0.822, *d* = 0.04), or between unhealthy food in motion (*M* = 2.70, *SD* = 1.09) and unhealthy static food (*M* = 2.52, *SD* = 1.13; *t*(244) = 1.15, *p* = 0.249, *d* = 0.2).

**Freshness perception (H4).** The effect of motion on freshness perception was not significant (*F*(1, 244) = 0.05, *p* = 0.821, *d* = 0.028). Participants did not perceive food in motion as more fresh (*M* = 6.14, *SD* = 1.88) than static food (*M* = 6.01, *SD* = 1.73). The effect of food category on freshness perception was significant (*F*(1, 244) = 262.72, *p* < 0.001, *d* = 2.057). Healthy food was perceived as more fresh (*M* = 7.44, *SD* = 0.94) than unhealthy food (*M* = 4.85, *SD* = 1.49). There was no significant interaction effect between motion and food category (*F*(1, 244) = 0.51, *p* = 0.475, *d* = 0.008). Perceived freshness did not differ between healthy food in motion (*M* = 7.52, *SD* = 1.04) and healthy static food (*M* = 7.37, *SD* = 0.81; *t*(244) = 0.65, *p* = 0.518, *d* = 0.119), or between unhealthy food in motion (*M* = 4.81, *SD* = 1.53) and unhealthy static food (*M* = 4.88, *SD* = 1.46; *t*(244) = 0.36, *p* = 0.721, *d* = 0.063).

**Anticipated pleasure of consumption (H5).** The effect of motion on anticipated pleasure was not significant (*F*(1, 244) = 0.31, *p* = 0.578, *d* = 0.071). Participants did not indicate a higher anticipated pleasure of consuming food in motion (*M* = 5.55, *SD* = 1.37) compared to static food (*M* = 5.44, *SD* = 1.38). The effect of food category on anticipated pleasure was significant (*F*(1, 244) = 27.70, *p* < 0.001, *d* = 0.668). Participants anticipated healthy food to be more pleasurable to consume (*M* = 5.94, *SD* = 1.15) than unhealthy food (*M* = 5.10, *SD* = 1.44). There was no significant interaction effect between motion and food category (*F*(1, 244) = 0.00, *p* = 0.968, *d* = 0). Anticipated pleasure did not differ between healthy food in motion (*M* = 5.96, *SD* = 1.18) and healthy static food (*M* = 5.93, *SD* = 1.12; *t*(244) = 0.41, *p* = 0.683, *d* = 0.075), or between unhealthy food in motion (*M* = 5.16, *SD* = 1.44) and unhealthy static food (*M* = 5.03, *SD* = 1.44; *t*(244) = 0.37, *p* = 0.708, *d* = 0.064).

**Effects of the covariates.** Participants’ HC had a significant effect on appeal (*F*(1, 244) = 10.30, *p* = 0.002, *d* = 0.407), taste perception (*F*(1, 244) = 11.12, *p* = 0.001, *d* = 0.423), and anticipated pleasure (*F*(1, 244) = 7.66, *p* = 0.006, *d* = 0.351). That is, the higher participants’ HC, the higher they rated the appeal (*r* = 0.17, *p* = 0.009), tastiness (*r* = 0.17, *p* = 0.006), and anticipated pleasure of consumption (*r* = 0.14, *p* = 0.024) of the food. Participants’ hunger level was not related to any dependent measure, but their thirst level significantly influenced anticipated pleasure (*F*(1, 244) = 6.34, *p* = 0.012, *d* = 0.319). The thirstier participants felt during the study, the higher they anticipated pleasure in consumption (*r* = 0.19, *p* = 0.003).

These findings again revealed non-significant results for all five main effects hypotheses (H1–H5; Table 5), replicating the null findings from Study 1 and failing to replicate the findings of Amar et al. [17], Gvili et al. [14,15], and Gvili et al. [16]. Similarly, we found that food in motion does not evoke a higher appeal (H1), taste perception (H2), healthiness perception (H3), or freshness perception (H4) compared to static food. Further, contrasting our expectations in H5, we did not find evidence for an effect of food in motion on anticipated pleasure of consuming the food.

Additionally, separate analyses for foods and drinks showed no significant effects of motion (vs. no motion) on any of the food perception variables, in line with the full analyses outlined here. However, there were marginally significant effects of drinks in motion (vs. static drinks) on appeal, freshness perception, and anticipated pleasure of consumption, in the hypothesized direction. Specifically, drinks in motion increased the appeal, perception of freshness, and anticipated pleasure of consuming the drink, compared to static drinks.

#### 4.5.2. Mediation and Moderated Mediation Analyses

**Mediation of freshness perception (H6).** According to the multilevel mediation analyses (model 4; [83]) with 10,000 Monte Carlo samples and 95% CIs, the effect of motion on freshness perception was not significant (*B* = 0.13, *SE* = 0.23, *t*(246) = 0.55, *p* = 0.581, *d* = 0.069). We found significant effects of freshness perception on appeal (*B* = 0.47, *SE* = 0.04, *t*(245) = 11.71, *p* < 0.001, *d* = 1.479), taste perception (*B* = 0.40, *SE* = 0.04, *t*(245) = 10.20, *p* < 0.001, *d* = 1.288), and healthiness perception (*B* = 1.19, *SE* = 0.06, *t*(245) = 21.63, *p* < 0.001, *d* = 2.731). The tests of mediation revealed no significant indirect effect of motion through freshness perception on appeal (ab = 0.06, *SE* = 0.11, 95% CI = (−0.1504; 0.2702)), taste perception (ab = 0.04, *SE* = 0.14, 95% CI = (−0.1315; 0.2357)), and healthiness perception (ab = 0.15, *SE* = 0.27, 95% CI = (−0.3805; 0.6925)). There were no significant remaining direct effects of motion on appeal (*B* = 0.12, *SE* = 0.14, *t*(245) = 0.85, *p* = 0.395, *d* = 0.107) taste perception (*B* = 0.04, *SE* = 0.14, *t*(245) = 0.30, *p* = 0.765, *d* = 0.038), or healthiness perception (*B* = 0.15, *SE* = 0.20, *t*(245) = 0.78, *p* = 0.439, *d* = 0.098). Among the covariates, HC was significantly related to appeal (*B* = 0.26, *SE* = 0.08, *t*(245) = 3.31, *p* = 0.001, *d* = 0.418) and taste perception (*B* = 0.26, *SE* = 0.07, *t*(245) = 3.53, *p* = 0.001, *d* = 0.446). Hunger level was marginally significantly related to taste perception (*B* = 0.08, *SE* = 0.05, *t*(245) = 1.75, *p* = 0.082, *d* = 0.221), and thirst level was not related to any dependent measure (*p* > 0.05).

These results again show no mediating effects of freshness perception for the effects of food in motion (vs. static food) on (a) appeal, (b) taste perception, or (c) healthiness perception. Similar to Study 1, these findings do not provide support for H6 nor replicate the results from prior studies on the underlying process of perceived freshness (Amar et al., 2021; Gvili et al., 2015a, 2015b, Gvili et al., 2017).

**Mediation of anticipated pleasure of consumption (H7).** According to the multilevel mediation analyses (model 4; [83]) with 10,000 Monte Carlo samples and 95% CIs, the effect of motion on anticipated pleasure was not significant (*B* = 0.12, *SE* = 0.17, *t*(246) = 0.72, *p* = 0.474, *d* = 0.091). We found significant effects of anticipated pleasure on appeal (*B* = 0.87, *SE* = 0.04, *t*(245) = 22.04, *p* < 0.001, *d* = 2.783), and taste perception (*B* = 0.90, *SE* = 0.03, *t*(245) = 35.85, *p* < 0.001, *d* = 4.527). The tests of mediation revealed no significant indirect effect of motion through anticipated pleasure on appeal (ab = 0.11, *SE* = 0.15, 95% CI = (−0.1862; 0.3990)), and taste perception (ab = 0.11, *SE* = 0.15, 95% CI = (−0.1953; 0.4076)). The remaining direct effects of motion on appeal (*B* = 0.08, *SE* = 0.10, *t*(245) = 0.74, *p* = 0.460, *d* = 0.093) and taste perception (*B* = −0.02, *SE* = 0.07, *t*(245) = −0.26, *p* = 0.797, *d* = −0.033) were not significant. Among the covariates, HC (*B* = 0.07, *SE* = 0.04, *t*(245) = 1.82, *p* = 0.071, *d* = 0.23) and thirst level (*B* = −0.07, *SE* = 0.02, *t*(245) = −2.81, *p* = 0.005, *d* = −0.355) were marginally significantly related to taste perception. Hunger level was not related to any dependent measure (*p* > 0.05).

Similar to Study 1, these findings did not provide support for H7. While anticipated pleasure of consuming food increases (a) appeal and (b) taste perception of the food, it does not mediate the effect of in motion (vs. no motion) on these food perceptions.

**Moderated mediation of FPO (H8).** We ran two multilevel moderated mediation analyses (model 7; [82]), with 10,000 Monte Carlo samples, 95% CIs, and the moderator FPO mean-centered at 5.29. The effect of FPO on anticipated pleasure was significant (*B* = 0.29, *SE* = 0.14, *t*(244) = 2.09, *p* = 0.038, *d* = 0.264), but the interaction effect of motion and FPO on anticipated pleasure was not (*B* = −0.31, *SE* = 0.19, *t*(244) = −1.63, *p* = 0.104, *d* = −0.206). There was no significant index of moderated mediation through anticipated pleasure on appeal (ab = −0.27, 95% CI = (−0.5914; 0.0501)) nor on taste perception (ab = −0.28, 95% CI = (−0.6189; 0.0578)).

Similar to what we found in Study 1, people with a higher (vs. lower) FPO anticipate more pleasure in consuming food. However, the interaction effects of motion and FPO on (a) appeal, and (b) taste perception are not mediated by anticipated pleasure of consumption. These results again failed to support H8.

## 5. Study 2b

Following the non-significant findings from Study 1–2a, this study was conducted as a final examination of the proposed effectiveness of food in motion. Study 2b was a replication of Study 2a but with different food stimuli. This study again examined the effects of food in motion on food perceptions (H1–H5), the mediation effects of freshness perception (H6) and anticipated pleasure (H7), as well as the moderated mediation effects of FPO (H8).

### 5.1. Stimuli and Pretest

The experimental stimuli for Study 2b were created by selecting a range of 15 relevant food pictures displaying implied motion of the food in online, publicly available photo databases (i.e., a variety of solid and liquid foods as well as healthy and unhealthy foods). To create the static food condition, we manipulated each motion picture similar to the prior studies.

We pretested the 15 manipulated pictures with the same aims as for Study 2a. We conducted an online pretest via our consumer panel with 71 participants (16% men, *M*_age_ = 35 years; *SD* = 12.00). The procedure was similar to the pretest from Study 1. Following a short introduction, pretest participants were presented with 15 randomized food pictures in the static condition. After exposure to each food picture, participants rated the perceived healthiness of the food and the appeal and realism of the picture on 9-point Likert scales (1 = Not at all, 9 = Very much). Afterward, participants filled out their gender and birth year.

For each food picture, we ran a series of three One-Sample T-Tests (one per measure), using the average value of the scale (i.e., 5) as the test value. We selected a total of 12 food pictures (Table A2), consisting of six foods that were perceived as healthy (*M* = 7.38, *SD* = 1.37; i.e., broccoli, bruschetta, lemon water, salad, orange juice, and chicken stew) and six foods that were perceived as unhealthy (*M* = 3.25, *SD* = 1.62; i.e., cheesecake, pancakes, profiteroles, ice cream, lime cake, and moelleux; Table 6). The food pictures were rated as sufficiently appealing (*M* = 6.95, *SD* = 1.83) and realistic (*M* = 7.25, *SD* = 1.63).

### 5.2. Participants

A total of 237 participants (23% men, *M*_age_ = 48 years; *SD* = 15.96) completed this study and were recruited online via our consumer panel. Similar to the previous studies, we used two attention checks in the experiment to screen the data after data collection. Specifically, we included an item in the FPO Scale, asking “*Please indicate ‘rather disagree’ for this statement*”, and another item in the HCS, asking “*Please indicate ‘rather agree for this statement*”. Twenty-one participants failed to answer these statements correctly and were excluded from the dataset. Our final sample included 216 participants, who were randomly assigned to one of four between-subjects conditions, similar to Study 2a: healthy food in motion (*n* = 52), unhealthy food in motion (*n* = 56), healthy static food (*n* = 54), or unhealthy static food (*n* = 54).

### 5.3. Procedure, Measurements, and Reliability

The procedure of this study was similar to Study 2a. After a short introduction, participants indicated their hunger and thirst levels on 7-point Likert scales (1 = Not hungry/thirsty at all, 7 = Very hungry/thirsty). Again, we included these covariates in our analyses.

Participants were then exposed to 6 randomized healthy or unhealthy food stimuli, presented either in motion or static, depending on the condition they were assigned to. As in the previous studies, after exposure to each picture, participants first rated the appeal, taste perception, healthiness perception, and freshness perception of the food presented in the picture (e.g., broccoli) on 9-point Likert scales (1 = Not at all, 9 = Very much). Secondly, anticipated pleasure of consumption was measured with a one-item 9-point Likert scale as in Study 2a, asking: “*How much pleasure do you anticipate in consuming this food [e.g., broccoli]?*” (1 = No pleasure at all, 9 = A lot of pleasure).

After stimulus presentation, we assessed participants’ FPO analogous to the prior studies, using the FPO Scale [50,76] on a 7-point Likert scale (1 = Strongly disagree, 7 = Strongly agree; *M* = 5.42; α = 0.73). Similarly, we assessed participants’ HC using the HCS [80] on a 7-point Likert scale (1 = Strongly disagree, 7 = Strongly agree; *M* = 4.98; α = 0.91) and included this score as a potential covariate in our analyses. Finally, participants filled out their gender and birth year.

### 5.4. Data Analysis

We ran multiple multilevel analyses taking the hierarchical data structure into account, using the REML estimation technique, VC as the default covariance structure, and estimated the intercept and slope randomly to account for participant-level and stimulus-level effects. Starting from fixed-effects models, model fit improved when we allowed for a random intercept, but it did not improve further when allowing for a random slope estimation.

The hypothesis testing for Study 2b is the same as for Study 2a. To test the main effects as proposed in H1–H5, we conducted five multilevel analyses with appeal, taste perception, healthiness perception, freshness perception, and anticipated pleasure as dependent measures respectively, motion (0 = Static food, 1 = Food in motion), food category (0 = Unhealthy food, 1 = Healthy food), and their interaction as independent variables, the nine food stimuli as the within-subjects variable, and participants’ HC, hunger, and thirst levels as covariates.

Furthermore, we ran three multilevel mediation analyses via MLmed [81], testing H6, with motion as the independent variable, freshness perception as the mediator, appeal, taste perception, and healthiness perception as dependent measures respectively, and participants’ HC and hunger and thirst levels as covariates. Additionally, we ran two multilevel mediation analyses via MLmed, to test H7, with motion as the independent variable, anticipated pleasure as the mediator, appeal and taste perception as dependent measures respectively, and participants’ HC, hunger, and thirst levels as covariates.

Finally, we ran two multilevel moderated mediation analyses via MLmed [82,83], to test H8. Motion served as the independent variable, FPO as the moderator, anticipated pleasure as the mediator, appeal and taste perception as dependent measures respectively, and participants’ HC, hunger, and thirst levels as covariates.

### 5.5. Results and Discussion

#### 5.5.1. Main Effects and Interaction Effects Analyses

**Appeal (H1).** The effect of motion on appeal was not significant (*F*(1, 209) = 1.58, *p* = 0.210, *d* = 0.172). Participants did not indicate a higher appeal of food in motion (*M* = 6.57 *SD* = 1.25) compared to static food (*M* = 6.39, *SD* = 1.29). The effect of food category on appeal was marginally significant (*F*(1, 209) = 3.49, *p* = 0.063, *d* = 0.255). There was a higher appeal of healthy food (*M* = 6.65, *SD* = 1.03) compared to unhealthy food (*M* = 6.31, *SD* = 1.45). There was no significant interaction effect between motion and food category (*F*(1, 209) = 0.00, *p* = 0.959, *d* = 0). There was no difference in appeal between healthy food in motion (*M* = 6.71, *SD* = 1.06) and healthy static food (*M* = 6.59, *SD* = 1.01; *t*(209) = 0.85, *p* = 0.400, *d* = 0.165), or between unhealthy food in motion (*M* = 6.44, *SD* = 1.40) and unhealthy static food (*M* = 6.19, *SD* = 1.51; *t*(209) = 0.93, *p* = 0.350, *d* = 0.177).

**Taste perception (H2).** The effect of motion on taste perception was not significant (*F*(1, 209) = 0.48, *p* = 0.491, *d* = 0.095). Participants did not perceive food in motion as tastier (*M* = 6.33, *SD* = 1.28) than static food (*M* = 6.27, *SD* = 1.33). The effect of food category on taste perception was significant (*F*(1, 209) = 8.28, *p* = 0.004, *d* = 0.393). Healthy food was perceived as tastier (*M* = 6.56, *SD* = 1.01) than unhealthy food (*M* = 6.05, *SD* = 1.49). There was no significant interaction effect between motion and food category (*F*(1, 244) = 0.00, *p* = 0.977, *d* = 0). Perceived taste did not differ between healthy food in motion (*M* = 6.55, *SD* = 1.07) and healthy static food (*M* = 6.57, *SD* = 0.96; *t*(209) = 0.50, *p* = 0.615, *d* = 0.097), or between unhealthy food in motion (*M* = 6.11, *SD* = 1.42) and unhealthy static food (*M* = 5.98, *SD* = 1.58; *t*(209) = 0.47, *p* = 0.637, *d* = 0.09).

**Healthiness perception (H3).** The effect of motion on healthiness perception was not significant (*F*(1, 209) = 0.12, *p* = 0.725, *d* = 0.047). Participants did not perceive food in motion as healthier (*M* = 5.09, *SD* = 2.29) than static food (*M* = 5.11, *SD* = 2.42). The effect of food category on healthiness perception was significant (*F*(1, 209) = 726.56, *p* < 0.001, *d* = 3.686). Not surprisingly, healthy food was perceived as healthier (*M* = 7.20, *SD* = 0.84) than unhealthy food (*M* = 3.08, *SD* = 1.36). There was no significant interaction effect between motion and food category (*F*(1, 209) = 0.79, *p* = 0.376, *d* = −0.001). Perceived healthiness did not differ between healthy food in motion (*M* = 7.12, *SD* = 0.88) and healthy static food (*M* = 7.27, *SD* = 0.81; *t*(209) = 0.37, *p* = 0.709, *d* = 0.072), or between unhealthy food in motion (*M* = 3.21, *SD* = 1.41) and unhealthy static food (*M* = 2.95, *SD* = 1.31; *t*(209) = 0.89, *p* = 0.376, *d* = 0.17).

**Freshness perception (H4).** The effect of motion on freshness perception was not significant (*F*(1, 209) = 1.05, *p* = 0.306, *d* = 0.14). Participants did not perceive food in motion as more fresh (*M* = 6.33, *SD* = 1.68) than static food (*M* = 6.62, *SD* = 1.76). The effect of food category on freshness perception was significant (*F*(1, 209) = 68.74, *p* < 0.001, *d* = 1.134). Healthy food was perceived as more fresh (*M* = 7.33, *SD* = 0.95) than unhealthy food (*M* = 5.64, *SD* = 1.88). There was no significant interaction effect between motion and food (*F*(1, 209) = 0.57, *p* = 0.452, *d* = −0.03). Perceived freshness did not differ between healthy food in motion (*M* = 7.23, *SD* = 0.96) and healthy static food (*M* = 7.44, *SD* = 0.93; *t*(209) = 0.19, *p* = 0.848, *d* = 0.037), or between unhealthy food in motion (*M* = 5.49, *SD* = 1.77) and unhealthy static food (*M* = 5.80, *SD* = 2.00; *t*(209) = 1.27, *p* = 0.204, *d* = 0.242).

**Anticipated pleasure of consumption (H5).** The effect of motion on anticipated pleasure was not significant (*F*(1, 209) = 1.90, *p* = 0.170, *d* = 0.188). Participants did not indicate a higher anticipated pleasure of consuming food in motion (*M* = 5.99, *SD* = 1.26) compared to static food (*M* = 5.79, *SD* = 1.47). The effect of food category on anticipated pleasure was significant (*F*(1, 209) = 19.12, *p* < 0.001, *d* = 0.598). Participants anticipated healthy food to be more pleasurable to consume (*M* = 6.29, *SD* = 1.13) than unhealthy food (*M* = 5.51, *SD* = 1.46). There was no significant interaction effect between motion and food category (*F*(1, 209) = 0.40, *p* = 0.529, *d* = 0.037). Anticipated pleasure did not differ between healthy food in motion (*M* = 6.31, *SD* = 1.05) and healthy static food (*M* = 6.27, *SD* = 1.21; *t*(209) = 0.52, *p* = 0.601, *d* = 0.101), or between unhealthy food in motion (*M* = 5.70, *SD* = 1.36) and unhealthy static food (*M* = 5.31, *SD* = 1.55; *t*(209) = 1.44, *p* = 0.152, *d* = 0.257).

**Effects of the covariates.** Participants’ HC had a significant effect on appeal *F*(1, 209) = 6.51, *p* = 0.011, *d* = 0.349), perceptions of taste (*F*(1, 209) = 5.62, *p* = 0.019, *d* = 0.037), healthiness *F*(1, 209) = 8.12, *p* = 0.005, *d* = 0.342), and freshness (*F*(1, 209) = 12.07, *p* = 0.001, *d* = 0.475). That is, the higher participants’ HC, the higher they rated the appeal (*r* = 0.18, *p* = 0.008), tastiness (*r* = 0.17, *p* = 0.011), healthiness (*r* = 0.14, *p* = 0.047), and freshness of the food (*r* = 0.23, *p* = 0.001). Participants’ hunger level was not related to any dependent measure. Participants’ thirst level, however, significantly influenced perceived taste (*F*(1, 209) = 7.33, *p* = 0.007, *d* = 0.37) and freshness (*F*(1, 209) = 4.05, *p* = 0.046, *d* = 0.275), as well as anticipated pleasure (*F*(1, 209) = 3.35, *p* = 0.068, *d* = 0.25). That is, the thirstier participants felt during the study, the higher they rated the taste (*r* = 0.17, *p* = 0.013), freshness (*r* = 0.11, *p* = 0.115), and anticipated pleasure of consumption (*r* = 0.12, *p* = 0.083) of the food.

These results again replicated the null findings as found in Study 1–2a for the effects of implied motion on food perceptions (H1–H5; Table 7).

Additionally, separate analyses for foods and drinks showed no significant effects of motion (vs. no motion) on any of the food perception variables, in line with the full analyses outlined here.

#### 5.5.2. Mediation and Moderated Mediation Analyses

**Mediation of freshness perception (H6).** According to the multilevel mediation analyses (model 4; [83]) with 10,000 Monte Carlo samples and 95% CIs, the effect of motion on freshness perception was not significant (*B* = −0.22, *SE* = 0.23, *t*(211) = −0.95, *p* = 0.341, *d* = −0.129). We found significant effects of freshness perception on appeal (*B* = 0.33, *SE* = 0.05, *t*(210) = 6.98, *p* < 0.001, *d* = 0.95), taste perception (*B* = 0.40, *SE* = 0.04, *t*(210) = 8.92, *p* < 0.001, *d* = 1.214), and healthiness perception (*B* = 0.94, *SE* = 0.07, *t*(210) = 13.09, *p* < 0.001, *d* = 1.781). The tests of mediation revealed no significant indirect effect of motion through freshness perception on appeal (ab = −0.07, *SE* = 0.08, 95% CI = (−0.2259; 0.0766)), taste perception (ab = −0.09, *SE* = 0.09, 95% CI = (−0.2703; 0.0912)), or healthiness perception (ab = −0.21, *SE* = 0.22, 95% CI = (−0.6455; 0.2338)). There were no significant remaining direct effects of motion on appeal (*B* = 0.29, *SE* = 0.16, *t*(210) = 1.83, *p* = 0.069, *d* = 0.249) taste perception (*B* = 0.20, *SE* = 0.15, *t*(210) = 1.36, *p* = 0.176, *d* = 0.185), or healthiness perception (*B* = 0.24, *SE* = 0.24, *t*(210) = 0.99, *p* = 0.324, *d* = 0.135). Among the covariates, only thirst level was significantly related to taste perception (*B* = 0.11, *SE* = 0.05, *t*(210) = 2.06, *p* = 0.041, *d* = 0.28). Participants’ HC and hunger level were not related to any dependent measure (*p* > 0.05).

Similar to the previous studies, we did not find mediating effects of freshness perception for the effects of motion (vs. no motion) on (a) appeal, (b) taste perception, or (c) healthiness perception, rejecting H6 and failing to replicate prior food in motion research.

**Mediation of anticipated pleasure of consumption (H7).** According to the multilevel mediation analyses (model 4; [83]) with 10,000 Monte Carlo samples and 95% CIs, the effect of motion on anticipated pleasure was not significant (*B* = 0.24, *SE* = 0.19, *t*(211) = 1.31, *p* = 0.191, *d* = 0.178). We found significant effects of anticipated pleasure on appeal (*B* = 0.68, *SE* = 0.04, *t*(211) = 15.56, *p* < 0.001, *d* = 2.117), and taste perception (*B* = 0.76, *SE* = 0.04, *t*(211) = 19.92, *p* < 0.001, *d* = 2.711). The tests of mediation revealed no significant indirect effect of motion through anticipated pleasure on appeal (ab = 0.17, *SE* = 0.13, 95% CI = (−0.0797; 0.4178)) or taste perception (ab = 0.19, *SE* = 0.14, 95% CI = (−0.0947; 0.4687)). The remaining direct effects of motion on appeal (*B* = 0.05, *SE* = 0.12, *t*(210) = 0.41, *p* = 0.682, *d* = 0.056) and taste perception (*B* = −0.07, *SE* = 0.10, *t*(210) = −0.67, *p* = 0.501, *d* = −0.091) were not significant. Among the covariates, HC was significantly related to appeal (*B* = 0.13, *SE* = 0.06, *t*(210) = 2.08, *p* = 0.039, *d* = 0.238) and marginally significantly related to taste perception (*B* = 0.10, *SE* = 0.05, *t*(210) = 1.90, *p* = 0.058, *d* = 0.259). Hunger level was not related to any dependent measure (*p* > 0.05). Thirst level was significantly related to taste perception (*B* = 0.07, *SE* = 0.04, *t*(210) = 2.01, *p* = 0.046, *d* = 0.274).

Similar to the previous studies, we did not find mediating effects of anticipated pleasure of consumption for the effects of motion (vs. no motion) on (a) appeal and (b) taste perception of the food, rejecting H7.

**Moderated mediation of FPO (H8).** We ran two multilevel moderated mediation analyses (model 7; [82]), with 10,000 Monte Carlo samples, 95% CIs, and moderator FPO mean-centered at 5.42. The effect of FPO on anticipated pleasure was significant (*B* = 0.42, *SE* = 0.14, *t*(209) = 3.05, *p* = 0.003, *d* = 0.415), but the interaction effect of motion and FPO on anticipated pleasure was not significant (*B* = −0.19, *SE* = 0.20, *t*(209) = −0.97, *p* = 0.334, *d* = −0.132). There was no significant index of moderated mediation through anticipated pleasure on appeal (ab = −0.13, 95% CI = (−0.3969; 0.1304)) nor on taste perception (ab = −0.14, 95% CI = (−0.4359; 0.1469)).

These findings again showed no moderated mediation effect of FPO on the effect of motion (vs. no motion) on (a) appeal and (b) taste perception through anticipated pleasure of consumption, thus rejecting H8.

## 6. Discussion

### 6.1. Overview of the Findings

This research investigated the effectiveness of using implied motion in food pictures on food perceptions through an anticipated pleasure of consumption in three experimental studies. We used a wide variety of food stimuli across multiple food categories, and different samples and study contexts, to test for the robustness and generalizability of our findings. Moreover, we used and manipulated existing food pictures to enhance external validity. Three major findings are discussed.

First, Studies 1, 2a, and 2b attempted to replicate the existing research on the effects of food in motion on appeal and perceptions of tastiness, healthiness, and freshness, as shown by Amar et al. [17], Gvili et al. [14,15], and Gvili et al. [16]. Across multiple food stimuli, we did not find an overall effect of using implied motion (vs. no motion) in food pictures on appeal and on perceptions of taste, healthiness, and freshness. Moreover, we did not find evidence for the underlying process of perceived freshness for the effectiveness of food in motion on food perceptions [14,15,16,17]. Worth mentioning is that we did find some limited significant effects for specific individual food stimuli per study, somewhat in line with the prior findings on the positive effects of food in motion [14,15,16,17]. However, we deliberately pooled multiple experimental stimuli to test for the robustness of the effects across a variety of food products, which is why we chose not to report these partial findings per stimulus for each study. There are several arguments for why we could not replicate the findings of [15,16,17]. For instance, their studies were most likely underpowered given the low average sample size per study, as indicated by two power analyses, based on G*Power [74] and a safeguard power analysis [75]. Following this, the original effects reported in [15,16,17] were probably overestimated, which could have led to biased conclusions. We showed in three studies with a larger sample size that the effects are not robust. Further, we used different experimental designs and stimuli from [15,16,17] as well as different statistical methods to analyze our data. The previous studies included only limited food categories and focused on a single food stimulus per experiment. Consequently, this could have led to a spontaneous positive finding for one individual food item, rather than consistent results across multiple and randomized food products, which those studies were not able to detect. By analyzing a large variety of experimental food stimuli with multilevel analyses, we were able to account for multiple data points for different food products per participant, which allows for more reliable and robust findings than only one data point. As such, as we find that the effects are highly dependent on the particular food product at display, our findings offer a more nuanced view on the supposed generalizability of the effects of using implied motion to visualize food products. Moreover, the experimental stimuli used in [15,16,17] displayed explicit implied movements in rather simple pictures without much detail, but there was no guarantee that the food pictures were sufficiently realistic, credible, or appealing. Contrastingly, the existing food pictures that were used (motion condition) and manipulated (static condition) in our studies were more detailed and highly suitable for advertising campaigns. We also pretested the manipulated pictures to make sure that they were appealing and realistic enough to be used in the studies. Hence, the manipulation of implied motion in the pictures of prior studies might have been noticed more or quicker by participants, which could have strengthened the effect of motion perception as found by [15,16,17]. The stronger motion is visualized in a food picture, the more likely it could be to find an effect of motion on food perceptions.

Secondly, we tested the proposed effectiveness of implied motion in pictures on food perceptions for both healthy and unhealthy food products in Study 2a–b. Following prior findings on food in motion [14,15,16,17], we did not anticipate particular differences between the two food categories, and that was also what we found. Neither food category showed differences in the effects of motion versus no motion on food perceptions. Notably, the findings did show that healthy food products were consistently rated as more appealing, tasty, fresh, and pleasurable to eat compared to unhealthy food products, regardless of whether the food was displayed in motion or not. This is remarkable considering the well-documented human tendency to favor unhealthy food over healthy food [19,20,48]. Possibly, social desirability bias might explain these findings. Study participants might have been more cognitively engaged and tried hard to do well on the experiment.

Thirdly, we aimed to extend the prior findings on motion perception in the food domain by investigating whether motion positively influences food perceptions through anticipated pleasure of consumption. Specifically, in Studies 1, 2a, and 2b, we examined the potential role of food pleasure as both a state variable (i.e., anticipated pleasure of consumption) and trait variable (i.e., food pleasure orientation) in relation to implied motion in food pictures. In contrast to our expectations, the use of motion in food pictures did not affect consumers’ anticipated pleasure of consuming the depicted food. Furthermore, this anticipated food pleasure did not serve as an alternative underlying process to freshness perception for the effect of motion on consumers’ appeal and tastiness perceptions of the food. Finally, although we found that people with a higher (vs. lower) FPO anticipate a higher pleasure of food consumption, this did not translate into higher appeal or taste perception of the food.

### 6.2. Theoretical and Practical Contributions

Our findings firstly contribute to the existing research on motion perception in the food domain. In light of the current replication crisis, we examined whether we could replicate the previous findings on the effectiveness of using implied motion in food pictures on food perceptions [14,15,16,17,65]. Essentially, we were not able to replicate these findings in three consecutive studies. The overall null findings across our studies seem to suggest that the use of implied motion in food pictures might not be a robust and reliable tool to enhance consumers’ appeal, tastiness, healthiness, and freshness perceptions of the depicted food. A relevant remark is that we did not conduct exact or direct replication studies, but rather conceptual replication or generalizability studies [73], as we used different methods, stimuli, and measures compared to Amar et al. [17], Gvili et al. [14,15], and Gvili et al. [16]. Yet, conceptual replications are also important as they help confirm whether the theoretical idea behind certain findings is true, and under which conditions those findings will or might occur. As such, these replication studies offer insights into how generalizable the findings are [73]. We believe that our proposed hypotheses are based on plausible theories that are well-grounded in motion perception research as well as studies on food perceptions. However, we find the expected effects only in a very limited number of stimuli. Consequently, we argue that it might be very stimulus- and context-dependent whether implied motion in food pictures can increase the appeal, tastiness, healthiness, or freshness perceptions of the depicted food. Hence, we reason that the proposed effectiveness of food in motion is weaker than previously indicated by [15,16,17]. Our findings indicate that the effects are not as robust as one might think based on their studies alone. The effectiveness of using implied motion in food pictures to increase food perceptions is limited and probably negligible for many food products and categories.

Further, we contribute to advertising research on healthy food promotion, by investigating a commonly used visual cue to display food. Previous research has shown that visual elements in food pictures can affect consumers’ responses to healthy and unhealthy food differently [6,34]. However, we find that implied motion does not impact consumer perceptions of healthy and unhealthy food products differently. Hence, we can reasonably assume that implied motion can be used in pictures to promote healthy foods, as this practice does not decrease relevant healthy food perceptions such as appeal and projected taste, although we show that it does not increase these perceptions either.

Moreover, we add to the growing body of literature on food pleasure. As the concept of food pleasure proves to be an efficient way of attracting consumers toward healthy food, it is crucial to find out how it can and cannot be triggered [12,50,51]. Following our findings, we exclude a plausible effect of implied motion on perceived appeal and tastiness of the food through anticipated pleasure of consuming the food. Although motion is a vivid cue that is more appealing than no motion [23], it might be rather subtle and thus not strong enough to affect consumers’ perceptions of the food. Additionally, we show that consumers’ food pleasure orientation does not strengthen the effect of implied motion on anticipated pleasure of consumption. We find that people with a higher versus lower food pleasure orientation have a higher anticipated pleasure of consuming the food they are viewing. According to Huang and Wu [50] and Rozin et al. [51], this increased FPO should translate into higher perceived tastiness of the food, which can diminish the healthy = less tasty intuition. However, we did not find such effects in our studies.

Our findings have a broader practical implication by pointing to the relevancy of thinking carefully about the product presentation in displays or advertising to create favorable food evaluations, especially for healthy food. While many marketers frequently use implied motion in their advertising to visualize foods, we show that the effectiveness of this practice is not straightforward. Our findings offer field practitioners a more nuanced assessment of the effectiveness of implied motion. This can help them in choosing the most beneficial visual elements in their food promotion pictures. Furthermore, keeping in mind the findings from our studies for visual advertising campaigns, promoting healthy food can be a valuable step in the right direction to increase healthy food consumption.

### 6.3. Limitations and Suggestions for Future Research

The implications of our findings are constrained by certain limitations, some of which can drive future research directions. First, the current research investigated consumer perceptions of food products in motion. However, actual buying and consumption behavior can differ from perceptions, attitudes, and intentions. Perception measures often fail to capture actual consumer behavior. Therefore, it might be valuable to measure the effect of implied motion (vs. no motion) in food pictures on food-related behavior such as behavioral intentions or actual food choices and consumption. For instance, future research could involve a field study in the context of online grocery shopping using pictures of food products in motion. Such experimental setups could test the ecological validity of the findings on the effectiveness of food in motion.

Further, while we intentionally examined implied motion of food with the aim of replicating the prior findings on food in motion, a relevant question is whether its effectiveness would differ for real motion (e.g., watching a video of someone pouring a drink rather than viewing a snapshot of this video). As neuroimaging studies show that exposure to implied motion activates similar regions of the brain as exposure to real motion [56,58,60,61,62], one might not expect striking differences between these two motion types. However, studies focusing on the effectiveness of real motion in food displays on pleasure and other food perceptions would increase the ecological validity of the findings, as in real life products, people, and animals are continuously moving around in the environment [84]. Particularly, many visual food advertisements are dynamic rather than static, consider videos, animated advertisements, animated GIFs (Graphics Interchange Formats), digital billboards, and so on. Interestingly, prior research in the animation context shows that animated ads increase arousal compared to static ones [23,24]. As such, dynamic motion in food advertisements could have stronger effects on pleasure and other food perceptions compared to the non-existing or weak effects of implied motion that we found. Following our reasoning that a stronger visualization of motion in a food display could lead to stronger effects on food perceptions, further research should investigate the different effects implied versus real motion can have on food-related behavior.

Similarly, different kinds of implied or real motion can exist within food displays, such as the distinction based on the subject that is making the movements. In this regard, motion is biological or animate when it comes from living organisms (e.g., a person sprinkling spices on a salad), but it can also be non-biological or inanimate, being from objects (e.g., a salad spinning on a plate). Interestingly, our visual attention system is more sensitive to biological motion than to non-biological motion [57,85,86]. Consequently, biological motion in food pictures might influence food perceptions more strongly than non-biological motion, an assumption worthy of future investigation. Similarly, a movement can occur on a food product (e.g., a picture of maple syrup dripping from a stack of pancakes), or around the product (e.g., a flowing motion of honey behind a stack of pancakes). Likewise, motion can be unrelated or unrealistic (e.g., a picture of a fruit drink with fruit slices splashing and flying in the air) as opposed to related or realistic (e.g., a picture of a fruit drink being poured into a glass).

Additionally, in the current studies, we focused on increasing the external validity of the findings on food in motion by using existing food pictures and a wide variety of healthy and unhealthy food stimuli across multiple food categories. However, a possible alternative way to manipulate healthy and unhealthy food in motion in future research is by using the same food picture and manipulating the healthiness perception with a written description (e.g., a salad with a dressing that is either described as being highly caloric, such as a cream and cheese dressing, or as being low in calories, such as a yogurt dressing). Such study designs and experimental stimuli would increase the internal validity of the findings. Moreover, rather than only measuring the proposed underlying process for the effectiveness of food in motion, i.e., the anticipated pleasure of consumption, an experimental manipulation of this mediator in future studies would further increase the internal validity of our findings.

Lastly, we took into account the potential moderation of food pleasure orientation for the effects of motion on anticipated pleasure. However, other moderators could also be of interest. For example, does a health (vs. pleasure) induced goal have a different impact on the effect of motion (vs. no motion) on food perceptions and behavior? Namely, consumers with a pleasure- (vs. health) oriented goal could be more drawn to the vividness of implied motion in a picture [23], which could lead to stronger effects of this visual cue on food perceptions. Similarly, while we included consumers’ health consciousness and their hunger and thirst states as relevant control variables in our studies, other state or trait variables might also be related to the effectiveness of food in motion. For instance, we did not include the hedonic liking of the foods and drinks as a control variable in our studies. However, anticipated pleasure of consumption as well as other food perception variables might be dependent on the relative liking of the food products. As such, future research should pretest food stimuli on the hedonic liking of the stimuli or include this variable as a covariate in the studies.

## 7. Conclusions

This research investigated the effectiveness of food in motion on food perceptions through an anticipated pleasure of consumption. While previous studies show that using implied motion in food can be an effective way to enhance the appeal, perceptions of taste, healthiness, and freshness, we did not find evidence for these effects across three experiments. Considering the current replication crisis, these findings provide more nuanced insights into how implied motion affects food perceptions. We further showed that food perceptions of healthy and unhealthy food in motion do not differ, although prior studies in food advertising suggest that visual cues can affect responses to healthy and unhealthy food differently. Finally, food in motion did not increase appeal or taste perceptions through a higher anticipated pleasure of consuming the food.

## Figures and Tables

**Table 1:** Overview of experimental studies.

Study	Experimental Design	Between-Subjects Conditions	Experimental Stimuli	Measurements
Study 1	Lab experiment (*N* = 159)	Food in motion vs. Static food	26 food pictures	Appeal Perceptions of taste, healthiness, and freshnessAnticipated pleasure of consumption
Study 2a	Online panel experiment (*N* = 251)	2 (Food in motion vs. Static food) X 2 (Healthy food vs. Unhealthy food)	9 healthy or unhealthy food pictures	Appeal Perceptions of taste, healthiness, and freshnessAnticipated pleasure of consumption
Study 2b	Online panel experiment (*N* = 216)	2 (Food in motion vs. Static food) X 2 (Healthy food vs. Unhealthy food)	6 healthy or unhealthy food pictures	Appeal Perceptions of taste, healthiness, and freshnessAnticipated pleasure of consumption

**Table 2:** Examples of the food stimuli used in Study 1.

	Healthy Food		Unhealthy Food	
**Food in motion**	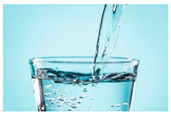	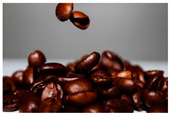	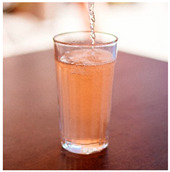	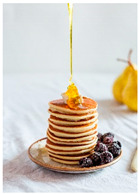
**Static food**	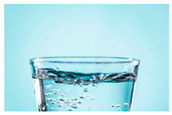	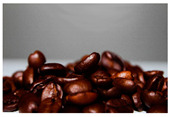	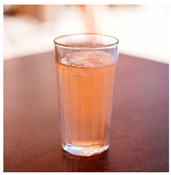	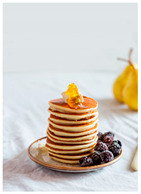

**Table 3:** Descriptives and multilevel results from the main effects in Study 1.

	Appeal		Taste Perception		Healthiness Perception		Freshness Perception		Anticipated Pleasure	
*Condition*	***M***	**(*SD*)**	***M***	**(*SD*)**	***M***	**(*SD*)**	***M***	**(*SD*)**	***M***	**(*SD*)**
Food in Motion	6.16	(0.65)	6.07	(0.68)	5.47	(0.55)	6.15	(0.71)	5.90	(0.71)
Static Food	6.00	(0.84)	5.99	(0.87)	5.54	(0.66)	6.05	(0.76)	5.81	(0.89)
	*F*(1, 154)									
**Motion**	1.48		0.29		0.74		0.45		0.23	
*Covariates*	*F*(1, 154)									
**HC**	5.12 *		1.00		0.75		1.31		1.50	
**Hunger Level**	0.01		0.91		0.22		0.00		3.33 ^(^*^)^	
**Thirst Level**	10.35 **		3.94 *		1.78		2.30		5.89 *	

Note: ^(^*^)^ *p* < 0.10; * *p* < 0.05; ** *p* < 0.01.

**Table 4:** Examples of the food stimuli used in Study 2a.

	Healthy Food		Unhealthy Food	
**Food in motion**	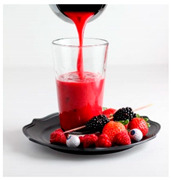	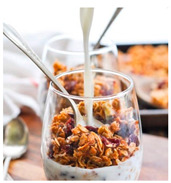	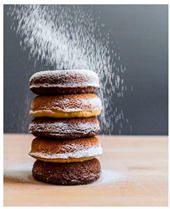	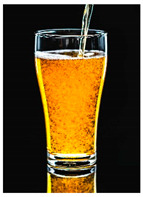
**Static food**	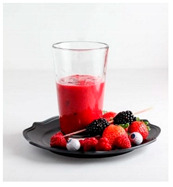	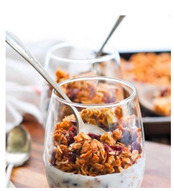	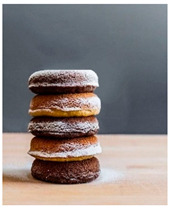	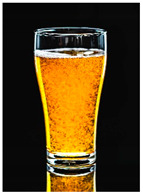

**Table 5:** Descriptives and multilevel results from the main effects in Study 2a.

	Appeal		Taste Perception		Healthiness Perception		Freshness Perception		Anticipated Pleasure	
*Condition*	***M***	**(*SD*)**	***M***	**(*SD*)**	***M***	**(*SD*)**	***M***	**(*SD*)**	***M***	**(*SD*)**
Food in Motion	6.13	(1.47)	5.78	(1.35)	5.12	(2.63)	6.14	(1.88)	5.55	(1.37)
Static Food	5.95	(1.43)	5.71	(1.34)	4.81	(2.69)	6.01	(1.73)	5.44	(1.38)
	*F*(1, 244)									
**Motion**	0.70		0.13		0.92		0.05		0.31	
*Covariates*	*F*(1, 244)									
**HC**	10.30 **		11.12 **		0.53		0.47		7.66 **	
**Hunger Level**	0.19		1.86		0.20		0.15		1.04	
**Thirst Level**	2.68		1.26		1.29		1.23		6.34 *	

Note: * *p* < 0.05; ** *p* < 0.01.

**Table 6:** Examples of the food stimuli used in Study 2b.

	Healthy Food		Unhealthy Food	
**Food in motion**	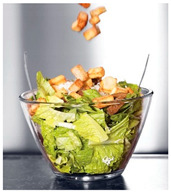	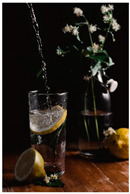	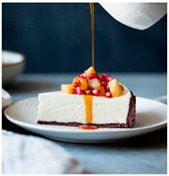	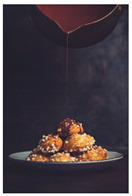
**Static food**	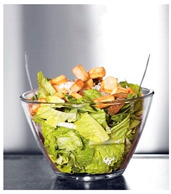	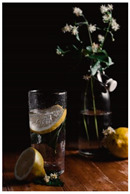	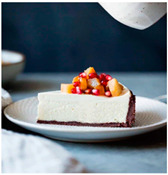	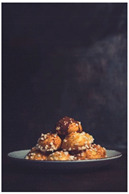

**Table 7:** Descriptives and multilevel results from the main effects in Study 2b.

	Appeal		Taste Perception		Healthiness Perception		Freshness Perception		Anticipated Pleasure	
*Condition*	***M***	**(*SD*)**	***M***	**(*SD*)**	***M***	**(*SD*)**	***M***	**(*SD*)**	***M***	**(*SD*)**
Food in Motion	6.57	(1.25)	6.33	(1.28)	5.09	(2.29)	6.33	(1.68)	5.99	(1.26)
Static Food	6.39	(1.29)	6.27	(1.33)	5.11	(2.42)	6.62	(1.76)	5.79	(1.47)
	*F*(1, 209)									
**Motion**	1.58		0.48		0.12		1.05		1.90	
*Covariates*	*F*(1, 209)									
**HC**	6.51 *		5.62 *		8.12 **		12.07 **		2.18	
**Hunger Level**	0.14		0.72		1.08		0.32		0.07	
**Thirst Level**	1.35		7.33 **		1.12		4.05*		3.35 ^(^*^)^	

Note: ^(^*^)^ *p* < 0.10; * *p* < 0.05; ** *p* < 0.01.

## Data Availability

The data presented in this study are available on request from the corresponding author. Although consumer data have been anonymized, data are not publicly available.

## Appendix A

**Table A1:** Overview of pretest results for the food stimuli used in Study 1 and Study 2a *.

Food Stimulus	Healthiness		Appeal		Realism	
	*M*	(*SD*)	*M*	(*SD*)	*M*	(*SD*)
Avocado salad with lime juice in bowl *	8.00	(1.01)	7.38	(1.48)	6.71	(1.69)
Brussels sprouts in colander *	8.11	(1.12)	6.03	(2.10)	5.32	(2.05)
Coffee beans	5.07	(1.97)	6.04	(2.06)	6.38	(1.82)
Fruit salad in bowl *	8.31	(0.96)	7.85	(1.38)	7.77	(1.38)
Herbal tea in cup *	7.57	(1.43)	6.25	(2.00)	6.50	(1.81)
Honey in bowl	5.11	(2.15)	5.87	(2.14)	5.83	(1.92)
Lemons cut on plate	7.98	(1.37)	7.39	(1.46)	7.76	(1.36)
Lettuce salad with dressing on plate	7.52	(1.45)	6.26	(1.95)	6.15	(1.87)
Lime squeezed	7.98	(1.14)	7.53	(1.52)	7.07	(1.88)
Milk in glass	6.73	(1.86)	5.07	(2.20)	6.55	(1.90)
Muesli with milk in bowl *	7.48	(1.50)	6.52	(1.75)	6.55	(1.64)
Muesli with milk in glass *	7.06	(1.60)	7.45	(1.40)	6.46	(1.77)
Olive oil in bowl	5.89	(1.84)	6.27	(1.78)	6.20	(1.95)
Orange juice in glass	5.62	(2.19)	6.20	(1.93)	7.57	(1.45)
Red berry smoothie in glass *	7.49	(1.51)	7.64	(1.47)	6.27	(1.88)
Tomato juice in glass *	7.80	(1.30)	5.77	(2.26)	6.31	(1.72)
Water in glass 1 *	8.56	(0.91)	7.07	(1.64)	6.78	(1.97)
Water in glass 2	8.15	(1.39)	6.12	(2.04)	6.22	(2.20)
Water in glass 3	8.12	(1.50)	6.84	(1.93)	6.11	(2.06)
Beer in glass 1 *	2.93	(1.64)	5.07	(2.52)	5.76	(2.06)
Beer in glass 2	2.80	(1.73)	5.25	(2.59)	5.31	(2.09)
Beer in glass 3	2.99	(1.45)	5.00	(1.94)	5.61	(2.28)
Cake with glaze on plate *	2.84	(1.22)	5.84	(2.21)	5.10	(2.02)
Chocolate cake with pink glaze on plate *	2.38	(1.30)	6.54	(1.90)	5.92	(1.80)
Coffee in mug	4.70	(1.85)	5.11	(2.27)	5.83	(2.01)
Coffee in thermos	4.36	(1.60)	5.27	(2.04)	6.28	(1.82)
Donuts with powdered sugar on plate *	2.46	(1.46)	6.42	(1.92)	5.24	(1.72)
Ice cream in cone *	2.46	(1.37)	6.65	(1.90)	6.84	(1.60)
Lemonade in glass *	4.37	(1.71)	5.14	(2.16)	6.61	(1.65)
Muffin with chocolate sauce *	2.43	(1.22)	5.89	(2.03)	6.56	(1.68)
Pancakes with honey on plate *	2.96	(1.37)	6.99	(1.93)	5.40	(1.97)
Pancakes with syrup *	2.11	(1.20)	7.07	(2.03)	5.09	(2.13)
Red grenadine in glass	3.69	(1.83)	5.28	(2.30)	4.98	(2.06)
Red wine in glass	3.63	(1.79)	5.67	(2.47)	5.53	(2.44)
Yellow grenadine in glass	3.49	(1.90)	5.47	(2.48)	5.41	(1.94)

Note: All stimuli were used in Study 1. Only the stimuli indicated with * were used in Study 2a.

**Table A2:** Overview of pretest results for the food stimuli used in Study 2b.

Food Stimulus	Healthiness		Appeal		Realism	
	*M*	(*SD*)	*M*	(*SD*)	*M*	(*SD*)
Broccoli with olive oil on plate	8.42	(1.07)	6.18	(2.04)	7.57	(1.57)
Bruschetta with balsamico on plate	6.71	(1.44)	7.94	(1.39)	7.85	(1.12)
Chicken stew with raisins in pot	6.78	(1.36)	7.08	(2.07)	7.08	(1.74)
Lemon water in glass	8.12	(1.34)	6.98	(1.82)	7.22	(1.97)
Lettuce salad with croutons in bowl	7.31	(1.29)	5.91	(1.90)	7.37	(1.31)
Orange juice in glass	6.94	(1.73)	7.56	(1.41)	7.78	(1.60)
Cheesecake with syrup on plate	4.55	(1.90)	7.64	(1.41)	7.40	(1.47)
Ice cream with caramel sauce in glass	2.76	(1.51)	6.82	(2.12)	6.91	(1.83)
Lime cake with glaze on plate	3.54	(1.45)	6.16	(2.23)	6.66	(1.64)
Moelleux with chocolate sauce	2.51	(1.57)	7.09	(1.93)	6.98	(1.89)
Pancakes with syrup on plate	3.67	(1.88)	7.89	(1.50)	7.75	(1.48)
Profiteroles with chocolate sauce on plate	2.49	(1.38)	6.20	(2.09)	6.38	(1.92)
